# Survey on Oral Health Education Knowledge of Family Members and Health Workers Dedicated to Patients with Disabilities

**DOI:** 10.1055/s-0043-1761188

**Published:** 2023-05-12

**Authors:** Francesco Saverio Ludovichetti, Andrea Zuccon, Patrizia Lucchi, Anna Giulia Signoriello, Edoardo Stellini, Sergio Mazzoleni

**Affiliations:** 1 Section of Dentistry, Department of Neurosciences, Università degli Studi di Padova, Padova, Italy

**Keywords:** Special Dentistry, health workers, oral hygiene, disabilities

## Abstract

**Objective** Over a billion people in the world live with some form of disability and this figure is destined to grow due to the increase in life expectancy. As consequence, the caregiver is taking on an increasingly important role that gains relevance also in the field of oral-dental prevention, being able to promptly identify needs for medical treatment. In some cases, however, the caregiver can be an obstacle to consider in case of a lack of adequate knowledge and commitment. The aim of this study is to evaluate the caregiver level of oral education comparing family members and health workers dedicated to people with disabilities knowledge.

**Materials and Methods** Anonymous questionnaires were distributed in five disability service centers and filled alternatively by family members of patients with disability and disability service centers health workers.

**Results** Two-hundred fifty questionnaires were collected, of which 100 were filled in by family members and 150 by health workers. Data were analyzed by applying the chi-squared (X2) independence test and the pairwise method for missing data.

**Conclusion** Family members oral education appears better in terms of brushing frequency, toothbrush replacement, and number of dental visits.

## Introduction

The World Health Organization (WHO) defines the “disability” as: “any limitation or loss (resulting from impairment) of the ability to perform an activity in the manner and extent considered normal for a human being; better still, all the disadvantageous conditions that affect humans determine a handicap.” 1

In Italy, there are approximately 3 million and 100 thousand disabled according to Istituto Nazionale di Statistica (ISTAT) data of 2016 (5.2% of the population). 2

Patients with special needs present sensory, mental, and motor alterations. Often, these symptoms appear in combination hindering mobility, language, mental development, learning, and self-sufficiency. 3

Special dentistry provides dental services to all those people with physical or cognitive conditions that limit their ability to receive healthcare. The goal is to allow them to be treated like able-bodied subjects by implementing special therapeutic strategies to carry out normal treatment plans. 4

In fact, the health of the mouth and teeth can have a great importance regarding the individual psychoemotional development since it reflects on the aesthetic function and on the level of self-esteem. In the disabled patient, this condition of well-being is essential to ensure, starting from a young age, a harmonious psychosomatic growth. 5

Empathy and respect are fundamental qualities of any good professional and can facilitate communication with the patient. 6

During personal hygiene treatments, many difficulties caused by different abilities of the patient can appear. This can result in less cooperation of the patient. There are many factors that may affect the collaboration of the disabled patient including the severity of mental retardation that can be assessed as total, partial, or zero based on the participation provided during dental treatment. The patient who has a mental retardation high enough to make the collaboration non-existent is defined as “non-cooperative.” 7

As far as oral disorders are concerned, patients with intellectual disabilities frequently suffer from caries and present periodontal disease at an early age due to difficulties in performing oral hygiene maneuvers. 8

These patients can often present malocclusions and oral malformations that cause difficulties in chewing, communication, and breathing. They may also have enamel defects, variations in the number, size, and shape of the dental elements; all of these anomalies can act as an obstacle to oral hygiene, increasing the risk of gingivitis, xerostomia, caries, and oral trauma. 9

Healthcare for people with special needs requires specialist knowledge, awareness, attention, and adaptation.

The “caregiver” is a person who takes care of the disabled person in his daily practical actions helping him in the management of the disease and in carrying out activities; they are usually family members. 10

The purpose of this study is to evaluate the level of oral hygiene education of family members, guardians, health workers and educators of patients with disabilities, and of all those involved in the management of the hygiene of the disabled patient.

## Materials and Methods

The data collection was performed from May 1 to September 30, 2022, by filling in paper questionnaires distributed in the disability service centers in the province of Treviso including day centers and some residential services. The questionnaires consisted of 21 multiple choices ( Supplementary Appendix A [online only]) and closed-ended questions chosen from current literature, and provided from 2 to 4 possible answers for each question.

The clinical sample used in the study was made up of family members, guardians, and parents of disabled children and social welfare and social–health workers who served in the dedicated centers. Two-hundred fifty questionnaires were collected, of which 100 were filled in by parents, family members, or guardians and 150 by social and healthcare / assistance health workers.

Regarding statistics, the chi-squared independence test (χ2) was used. This test is applied to analyze the existence or not of an association between the variables that were considered in the construction of the contingency table obtained from the research data.

## Results

Concerning the variable “Has the patient already experienced dental caries?” in Table 1 , we realize that most of the patients have already suffered from carious pathology and, among those who had it, most were assisted by “health workers.” Regarding the independence test, we observed that the *p* -value is less than 0.05, and therefore the presence of caries can be influenced by being assisted by “health workers” rather than by “parents / family members.” Furthermore, it can be inferred that those who are cared for by a parent/family member have an 8.52 times lower chance of having caries than those who are cared for by a health workers.

**Table 1:** Frequency distribution of response according to “Has the patient already experienced dental caries?”

Role	Has the patient already experienced dental caries?	
	No	Yes
Family member	15 (6.05%)	85 (34.27%)
Health worker	03 (1.21%)	145 (58.47%)
Chi-squared test, *p* -value = 0.00011212		

In Table 2 , the variable “If the patient is not able to brush his teeth independently, do you personally take care of brushing his teeth?” was investigated. It has been observed that the majority replied that they take care of brushing the patient's teeth, in case he is not able to do it independently. About 62.5% of those who hold the role of parent/family member brush the patient's teeth when unable, while that percentage was 84.56% in case of health workers. Regarding the verification of independence, we observed that the *p* -value was less than 0.05, and therefore we reject the null hypothesis, that is, there is a dependence between the variables. It can also be seen that the possibility that the parent/family member does not take care of brushing the patient's teeth is 3.28 times greater than for health workers.

**Table 2:** Frequency distribution of response according to “If patients are not able to brush his teeth independently, do you personally take care of brushing their teeth?”

Role	If the patient is not able to brush his teeth independently, do you personally take care of brushing his teeth?	
	No	Yes
Family member	36 (14.69%)	60 (24.49%)
Health worker	23 (9.39%)	126 (51.43%)
Chi-squared test, *p* -value = 8.0523e-05		

In Table 3 , it was observed that the *p* -value is lower than the established significance level (5%) and therefore we will reject the null hypothesis. So, there is an influence between the variables. Most of the patients brush their teeth twice a day, whether they are assisted by parent/family or by health workers. Despite this, those who are followed by a parent/family member are 1.72 more likely to brush their teeth twice a day instead of once.

**Table 3:** Frequency distribution of response according to “How many times per day are the teeth brushed?”

Role	How many times per day are the teeth brushed?			
	Once	Twice	3 times	never
Family member	21 (8.64%)	41 (16.87%)	33 (13.58%)	01 (0.41%)
Health worker	53 (21.84%)	60 (24.69%)	26 (10.70%)	08 (3.29%)
Chi-squared test, *p* -value = 0.003533				

In Table 4 , we can see that among those who hold the role of parent/family member, 38.14% change the brush every 3 months, while for the health workers this percentage was 37.58%. About 48.32% of those who hold the role of health workers, on the other hand, change the brush only when it presents some deformity. The *p* -value of the test performed was less than 0.05 and therefore there is a dependency between the variables. In fact, we can see that most of those who are cared for by a parent/family member are 2.37 times more likely to change their toothbrush every 2 months than those who are cared for by a care provider, who are more likely to change brush every 3 months.

**Table 4:** Frequency distribution of the response to “How often is the toothbrush changed?”

Role	How often is the toothbrush changed?”		
	Every 3 months	Every 2 months	When it presents deformities
Family member	37 (15.04%)	33 (13.41%)	27 (10.98%)
Health worker	56 (22.76%)	21 (8.54%)	72 (29.27%)
Chi-squared test, *p* -value = 0.00022			

Continuing the analyses, in Table 5 it is reported the variable “Do you think daily oral hygiene is responsibility of”: and as for the previous results, based on the *p* -value, we reject the hypothesis that the variables are independent. We can also observe that the response “to the dentist and the hygienist who takes care of him” has been very low. It is highlighted that most parents think that daily oral hygiene is the responsibility of family members; conversely most of the health workers think that it is up to the health workers themselves.

**Table 5:** Frequency distribution of the response to “Do you think that daily oral hygiene is the responsibility of”?

Role	Pensi che l'igiene orale quotidiana spetti:		
	Health workers	Family members	Dentist/dental hygienist
Family member	10 (5%)	62 (31%)	8 (4%)
Health worker	84 (42%)	33 (16.5%)	3 (1.5%)
Chi-squared test, *p* -value = 1.3106e-14			

Table 6 shows the opinion of the parents/family members and health workers who replied that the frequency of the dental visit must be at least once a year. It can also be highlighted how the probability that an individual assisted by a parent/family member will undergo a dental visit several times a year is 1.47 times greater compared with patients assisted by a health worker.

**Table 6:** Frequency distribution of the response to “how often do you think the checkup/dental visit should be done?”

Role	How often do you think the checkup / dental visit should be done?”			
	Once a year	More than once a year	When it is needed	Every month
Family member	52 (21.22%)	26 (10.61%)	16 (6.53%)	4 (1.63%)
Health worker	97 (39.59%)	33 (13.47%)	15 (6.12%)	2 (0.82%)
Chi-squared test, *p* -value = 0.00022				

In Tables 7 and 8 , the estimated variables are “Did you receive information on the prevention of caries and oral diseases?” and “Do you find the idea of practicing oral hygiene maneuvers on a third person unpleasant?” Contrary to the other results, for both variables the test was found to be not significant, that is, the *p* -value is greater than the established significance level. Therefore, the null hypothesis is not rejected, so there is no influence on either variable.

**Table 7:** Frequency distribution of the response to “Did you receive information on the prevention of caries and oral diseases?”

Role	Did you receive information on the prevention of caries and oral diseases?	
	No	Yes
Family member	41 (16.94%)	56 (23.14%)
Health worker	60 (24.79%)	85 (35.12%)
Chi-squared test, *p* -value = 0.8907		

**Table 8:** Frequency distribution of the response to “Do you find the idea of practicing oral hygiene maneuvers on a third person unpleasant?”

Role	Do you find the idea of practicing oral hygiene maneuvers on a third person unpleasant?”	
	No	Yes
Family member	62 (26.05%)	28 (11.76%)
Health worker	114 (47.90%)	34 (14.29%)
Chi-squared test, *p* -value = 0.1654		

## Discussion

Over one billion people around the world live with some form of disability. 1 Therefore, the caregiver takes a relevant position, but, sometimes, can represent an obstacle to care due to the lack of knowledge, unawareness, and commitment that this role requires. 11 Caregivers, educators, and social health and welfare health workers cover decisive roles regarding non-self-sufficient patients and take on a not insignificant importance in the field of oral-dental prevention as they can promptly identify any need for medical treatment. The aim of the study is to understand whether family members, guardians, health workers, and educators of patients with disabilities possess adequate basic knowledge of oral hygiene.

In relation to the question “Has the patient already experienced dental caries?,” it is inferred that most of the subjects with previous experiences of dental caries were assisted by social and health workers, assistance, and educators, while it is observed that subjects assisted by parents and/or family members are less likely to have been afflicted by caries pathology than to those who have been followed by a health worker. It follows that the presence or absence of dental caries could be influenced by being assisted by health workers and educators rather than by parents and family members; or it could simply depend on the fact that the health workers answered a question referring to all their patients (dealing with several subjects daily). In this way, the chances of finding at least one subject affected by dental caries in a single center are much higher than the possibility of finding the same pathology in the only patient examined. Many authors have studied the oral-dental health of non-self-sufficient patients and, while all studies agree on the prevalence of poor oral hygiene in individuals with disabilities, 12 13 the data from caries disease studies are often divergent. Some studies support the theory of a higher caries rate in individuals with disabilities than in the general population 14 15 ; other works report a lower incidence of caries in people with disabilities than in able-bodied individuals 16 ; still others do not report significant differences. 17 18

As for the question “If the patient is unable to brush their teeth independently, do you personally take care of brushing their teeth?,” we can see that most of the participants in the questionnaire help the patient in oral hygiene maneuvers when he is unable to do it independently, regardless of whether they are health workers or family members. These data coincide with the study of Petrovic et al, who argues that most people with developmental disabilities receive assistance and that more than 50% of these nonfamily auxiliaries are professional assistants including service providers and coordinators. 19 Most of the positive answers to the question came from health workers. It can, therefore, be deduced that health workers are more likely to support the patient during oral hygiene maneuvers than family members. In conclusion, the probability that the family member is in charge of brushing the teeth of the patient is about three times lower than the probability that a health worker may have.

As for the question “how many times a day are teeth brushed?” we can deduce that almost all subjects brush their teeth at least twice a day, but that the probability of brushing their teeth twice a day rather than just once is greater when they are assisted by a family member. The results of our study agree with that of Petrovic et al in which it was found that concomitant institutionalization and disability are significantly associated with an increased likelihood of developing gingivitis. 20

When asked “how often is the toothbrush changed?” we noticed that the tendency is to change the toothbrush about every 3 months, while a slightly lower part changes the toothbrush only when the bristles have deformed. Shah in 2017 recorded similar data in his study conducted in Alkharj, Saudi Arabia. Taking only the health workers as a reference, in fact, we can find the similarity of the data to the question about the useful life of a toothbrush. More than 40% of the interviewees (belonging to the category of health workers) stated “until the bristles are deformed.” 21

To the question “do you think oral hygiene belongs to” the answer “to the dentist and hygienist who take care of it” was very uncommon, while it is highlighted that most of the interviewees, both health workers and family members, consider that daily oral hygiene is their responsibility. Close collaboration between family members, health workers, and specialized health professionals can increase the well-being of the non-self-sufficient patient and delay invasive interventions.

With reference to the question relating to the frequency of the dental check-up/visit, it is possible to deduce that most of the participants in the study believe that a dental visit should occur at least once a year; however, the chances that those assisted by a family member are subjected to a dental visit more than once a year are higher than those assisted by a health worker. Attendance at dental appointments in line with recommendations is low worldwide. 22

As regards the question “have you received information on the prevention of caries and oral diseases?,” 16.94% for family members and 24.79% for health workers assert that they have not received any information on the prevention of caries and diseases oral. Increasing the promotion of oral health, the prevention of oral diseases and oral-dental health education is essential to reduce the barriers that people with intellectual disabilities and low socioeconomic conditions encounter when they require oral health treatments. 23 As far as the maintaining the oral health of people with special needs is concerned, Glassman and Subar demonstrated that caregiver training can have a positive impact on the oral health of people with developmental disabilities living in community settings. 11

Finally, analyzing the question “Do you find the idea of practicing oral hygiene maneuvers unpleasant?” a large number answered yes in agreement with the study of Cumella et al in which it emerged that some assistants expressed dislike for tooth brushing, which could have made them reluctant to address the dental needs of their clients.

## Conclusion

The results of our study showed that the level of oral education of the family member is higher than that of the health worker, probably due to the fact that the patient receives more care as the frequency of oral hygiene is greater over the course of a day, the toothbrush is replaced more frequently, and follow-up visits are shorter. Training of health workers is important to improve the knowledge of patients' needs and the ability of health workers to work effectively with these people. The associations and organizers of reception centers should promote and prioritize staff training to ensure that health workers acquire an adequate and up-to-date knowledge. Furthermore, the close collaboration between health workers and specialist health professionals can be decisive for the well-being of these fragile subjects.
