# Supplementary material for: Survey on Oral Health Education Knowledge of Family Members and Health Workers Dedicated to Patients with Disabilities
Source: Eur J Dent. 2023 May 12;17(4):1325–9. doi: 10.1055/s-0043-1761188 (PMC10756851; doi:10.1055/s-0043-1761188)
Supplement: Supplementary file 1 — Supplementary Material [file 10-1055-s-0043-1761188-s22102433.pdf]

## Supplementary Appendix A

1) What role do you have toward the boy/girl?
 a) Parent/family member
 b) Health worker

2) Do you think dental caries is a transmissible disease?
 a) Yes
 b) No
 c) I don't know

3) Do you believe in the possible development of a vaccine against dental caries?
 a) Yes
 b) No
 c) I don't know

4) Has the patient already experienced dental caries?
 a) Yes
 b) No

5) Has the patient already experienced gingivitis?
 a) Yes
 b) No

6) If the patient is not able to brush his teeth independently, do you personally take care of brushing his teeth?
 a) Yes
 b) No

7) How many times a day are the teeth brushed
 a) Never
 b) 1
 c) 2
 d) 3

8) What kind of toothbrush is used?
 a) Manual
 b) Electric

9) The toothbrush used is
 a) Soft
 b) Medium
 c) Hard
 d) I don't know

10) What kind of toothpaste is used?
 a) None
 b) Children's toothpaste
 c) Adult's toothpaste

11) How often the toothbrush is changed?
 a) Less than 2 months
 b) Every 3 months
 c) When the bristles are deformed

12) Is the mouthwash used?
 a) Yes
 b) No

13) Do you think tongue hygiene is important?
 a) Yes
 b) No

14) If you notice blood while teeth brushing
 a) I stop or I say to stop because it is hurting
 b) I continue, paying more attention

15) Is dental floss or an interdental brush used?
 a) Every day
 b) Once per week
 c) Once per month
 d) Never

16) Do you think daily oral hygiene is responsibility of:
 a) Family members
 b) Health workers
 c) To the dentist and the hygienist who takes care of him

17) How often do you think a dental visit should be performed?
 a) At least once per year
 b) More than once per year
 c) When it is needed
 d) Once per month

18) How many times a day sugary foods or drinks are consumed?
 a) Never
 b) 1 to 3 times per day
 c) More than 3 times per day

19) Did you receive information on the prevention of caries and oral diseases?
 a) Yes
 b) No

20) If yes, by whom?
 a) General doctor
 b) Doctors in the health center
 c) Dentist
 d) Other

21) Do you find the idea of practicing oral hygiene maneuvers on a third person unpleasant?
 a) Yes
 b) No

 © 2023. The Author(s).
